# Oncolytic viruses combined with immune checkpoint therapy for colorectal cancer is a promising treatment option

**DOI:** 10.3389/fimmu.2022.961796

**Published:** 2022-07-15

**Authors:** Yi Ren, Jia-Meng Miao, Yuan-Yuan Wang, Zheng Fan, Xian-Bin Kong, Long Yang, Gong Cheng

**Affiliations:** ^1^ College of Traditional Chinese medicine, Tianjin University of Traditional Chinese Medicine, Tianjin, China; ^2^ Research Center for Infectious Diseases, Tianjin University of Traditional Chinese Medicine, Tianjin, China; ^3^ School of Integrative Medicine, Tianjin University of Traditional Chinese Medicine, Tianjin, China; ^4^ Department of Critical Medicine, The First Affiliated Hospital of Suzhou University, Suzhou, China; ^5^ Tsinghua-Peking Joint Center for Life Sciences, School of Medicine, Tsinghua University, Beijing, China; ^6^ Institute of Infectious Diseases, Shenzhen Bay Laboratory, Shenzhen, China

**Keywords:** oncolytic viruses, immune checkpoint inhibitor, colorectal cancer, tumor microenvironment, immunotherapy

## Abstract

Immunotherapy is one of the promising strategies in the treatment of oncology. Immune checkpoint inhibitors, as a type of immunotherapy, have no significant efficacy in the clinical treatment of patients with pMMR/MSS/MSI-L mCRC alone. Therefore, there is an urgent need to find combination therapies that can improve the response rate of immune checkpoint inhibitors. Oncolytic viruses are a new class of cancer drugs that, in addition to directly lysing tumor cells, can facilitate the action of immune checkpoint inhibitors by modulating the tumor microenvironment and transforming “cold” tumors into “hot” ones. The combination of oncolytic viruses and immune checkpoint inhibitors is currently being used in several primary and clinical studies to treat tumors with exciting results. The combination of genetically modified “armed” OV with ICIs is expected to be one of the treatment options for pMMR/MSS/MSI-L mCRC. In this paper, we will analyze the current status of oncolytic viruses and ICIs available for the treatment of CRC. The feasibility of OV in combination with ICI for CRC will be discussed in terms of the mechanism of action of OV in treating tumors.

## Introduction

Colorectal cancer (CRC) is one of the most common and deadly cancers in the world community. It is the second most common cancer in women and the third most common in men. In 2018, there were 1.8 million CRC cases and 880,792 deaths worldwide, and CRC incidence is increasing in people under 50 years of age (1). The standard conventional treatments for CRC are surgery, chemotherapy, and radiotherapy, but because these three approaches have therapeutic limitations, immunotherapy has emerged as one of the newer options for treating CRC. In contrast to standard treatments, immunotherapy uses the patient’s immune system to fight cancer cells by modulating the innate and adaptive immune response, which overcomes the problem of specificity in tumor treatment and provides a further breakthrough in the treatment of CRC (2).

Immune checkpoint inhibitors (ICIs) are one of the popular immunotherapies for treating tumors in recent years, and they have shown exciting results in treating melanoma (3). Immune checkpoint inhibitors (ICIs) are monoclonal antibodies that activate the immune response by targeting and inhibiting immune checkpoints, including CTLA-4 and PD-1. Currently, ICIs have shown promising results in the treatment of mismatch-repair-deficient (dMMR) or high levels of microsatellite instability (MSI-H) (dMMR-MSI-H) CRC. However, ICIs have no benefit for treatment mismatch-repair-savvy (pMMR) and microsatellite stable (MSS) or low levels of microsatellite instability (MSI-L) (pMMR-MSI-L) CRC (4). Therefore, there is an urgent need to find combination therapies that can increase the response rate of pMMR/MSS/MSI-L mCRC to immune checkpoint inhibitors.

Oncolytic viruses (OVs) are a novel class of tumor therapeutic strategy to reduce tumors through preferential replication in tumor cells and stimulate host anti-tumor immunity to promote the lysis of tumors. There are many DNA and RNA viruses that can be used as OVs. At present, the viruses most commonly used in experimental cancer research are poxviruses, reoviruses, herpes simplex viruses (HSV), and adenovirus (5). The therapeutic activity of OVs is not limited to its tumor lytic activity but also includes the integrated modulation of the tumor microenvironment (TME) and immune system (6). Thus, OVs provide an ideal means to reverse immunosuppression of the tumor microenvironment and sensitize the tumor to immune checkpoint blockade (7). In addition, transgenes can be inserted into the OV genome through viral genome engineering techniques, where virally encoded gene expression can immunomodulate tumors. New OVs expressing checkpoint inhibitory antibody molecules can also be created. Through viral antibody therapy, i.e., genetic delivery of recombinant antibodies, combined with different modes of direct and indirect cancer cell killing, it can enhance the therapeutic efficacy, reduce the chance of drug resistance, and increase the local immune response to tumor cells (8).

With the marketing approval of the first oncolytic virus, Talimogene laherparepvec (T-VEC), for the treatment of advanced melanoma, oncolytic virus immunotherapy has been used in routine clinical oncology research (9). The combination of OVs with other immunotherapies, particularly with immune checkpoint inhibitors (ICIs), has shown promise as a treatment for oncology. The combination of OVs and ICIs could have a synergistic effect in increasing the response rate of tumor cells to immunotherapeutic agents, which may play an essential role in the future of clinical cancer treatment (10). This paper will analyze the oncolytic viruses that can be used to treat CRC and the current situation of ICIs in the treatment of CRC and discuss the feasibility of OVs combined with ICIs in the treatment of CRC from the mechanism of OVs in the treatment of tumor.

## Oncolytic virus for treating colorectal cancer

### Vaccinia virus

Vaccinia virus (VV) has a safety profile in humans as a smallpox vaccine. Recombinant VV has been used as an expression vector to enhance the tumor lytic effect and has been widely used in clinical trial studies in tumor models and patients with advanced solid cancers (11).

Researchers (12) constructed an attenuated strain of VG9 containing IL-24 (VG9-IL-24) targeting vaccinia virus. VG9-IL-24 induced specific and durable immune responses against colorectal tumors, produced enhanced killing of CRC cells, and inhibited the growth of colorectal cancer tumors, including through the induction of apoptosis in CRC cells *via* multiple apoptotic signaling pathways.

Colorectal cancer (CRC) frequently causes the spread of tumor cells within the peritoneal cavity, eventually leading to peritoneal carcinoma (PC) (13). Peritoneal metastases (PM) occur in about a quarter of CRC patients, and peritoneal metastases from CRC are considered to be the end-stage of the disease, second only to liver and lung metastases in terms of incidence, with a poor prognosis and one of the leading causes of patient death (14). A research team found that a GM-CSF carrying tumor lysing vaccinia virus JX-594 (pexastimogene devacirepvec, Pexa-Vec) effectively inhibits CRC peritoneal metastasis by selectively infecting and lysing peritoneal tumor cells and activating peritoneal dendritic cells (DCs) and CD8 T cells to restore peritoneal anti-cancer immunity (15).

### Reoviruses

Oncolytic reovirus (pelareorep) is a non-enveloped dsRNA virus that selectively lyses KRAS-mutated colorectal tumor cells. KRAS mutations are prevalent in 40-45% of colorectal cancer (CRC) patients, and treatment options are limited. One study found that (16), pelareorep preferentially induces autophagic mechanisms by up-regulating several vital autophagic proteins in KRAS mutations, which further triggers the apoptotic pathway, resulting in increased apoptosis and cell death in CRC cells. Meanwhile, it has also been demonstrated that (17) In CRC patients with KRAS mutations, pelareorep enhances immune efficacy and exerts tumor lytic effects by up-regulating the expression of surface peptides of MHC I molecules and activating CD4 and CD8 T cell populations.

### HSV

Herpes simplex viruses can be classified into types I and II (HSV-1, HSV-2 for short). Related researchers have genetically modified HSV-1 to express IL-12. HSV-1 expressing IL-12 promotes the cytolytic activity of natural killer cells and cytotoxic T lymphocytes, infects and kills colorectal cancer cell lines, and promotes anti-tumor immunity (18). oHSV-2 has been shown to alter the tumor microenvironment (TME) by increasing the infiltration of immune cells (NK cells, CD8 cells, and DCs), leading the TME from an immunosuppressed state to an anti-tumor immune state, thus transforming a “cold” tumor into a “hot” one (19). Meanwhile, there is experimental evidence that (20) oHSV-2 has been shown to have anti-tumor activity in patients with metastatic rectal cancer, with better efficacy when oHSV-2 is combined with immune checkpoint inhibitors.

### Adenovirus

Adenovirus is the more commonly used oncolytic virus vector, and the genome can be easily genetically modified to achieve better therapeutic results (21). At present, there are many genetically modified oncolytic adenoviruses used in the treatment of colorectal cancer. Researchers have constructed an oncolytic adenovirus (SPDD-UG) carrying SNORD44 (a C/D box snoRNA) and the GAS5 gene, which has been shown to inhibit the growth of colorectal cancer cells and induce apoptosis (22). In addition, an oncolytic adenovirus (rAd.DCN.GM) combining core proteoglycans and expressing granulocyte-macrophage colony-stimulating factor (GM-CSF) was shown to inhibit the growth and distant metastasis of colorectal cancer cells (23). In addition, a novel dual-targeted oncolytic adenovirus CD55-TRAIL combined with luteolin inhibited CRC cell proliferation while minimizing cytotoxicity to normal cells (24). A related experimental study found that adenovirus from a Non-human apes (GAd) encoding multiple neoantigens resulted in a novel GAd that effectively controlled tumor growth in mice and had a high potential for tumor eradication when combined with ICIs (25). Although the combination of adenovirus and ICIs has not yet been applied to colorectal cancer, there is still great promise in modifying adenovirus in various ways and using it in combination with ICIs to treat colorectal cancer.

### Other viruses

An oncolytic measles virus (OMV) encoding IL-12 can effectively induce apoptosis in rectal colon cancer cells by activating the IL-12/IFN-c/TNF-a inflammatory response (26). Orf virus (ORFV) can rapidly mediate innate and adaptive immune responses *in vivo*, so it has been proposed as a potential oncolytic virus vector. Studies have demonstrated that ORFV strain NA1/11 can inhibit CRC growth and metastasis by inducing apoptosis in CRC cells (27). In addition, a research team (28) produced M51R genetically engineered recombinant virus from cDNA clones containing the M protein mutation to construct M51R vesicular stomatitis virus (M51R VSV), which inhibits CRC peritoneal surface spread (PSD). Combined with immune checkpoint inhibitors may enhance the oncolytic potential of M51R VSV by reversing the immunosuppressive microenvironment of the peritoneum. Coxsackievirus B3 (CVB3) strain PD effectively infected and lyse CRC cells in a mouse model of colorectal cancer and demonstrated a good safety profile (29). On this basis, the researchers generated the cDNA clone of CVB3 variant PD-0 and generated the recombinant CVB3 variant PD-H from the clone. In immunocompetent mice, PD-H showed potent oncolytic activity against colorectal cancer and prevented the development of tumor malignancy (30) **(** **Table 1** **)**.

**Table 1:** Oncolytic virus available for colorectal cancer treatment.

Virus	Type of virus	Route of virus administration	Effect	References
VV	VG9-IL-24	i.t./i.p.	Significantly inhibits tumour growth and induces apoptosis in colorectal cancer cells	(12)
	JX-594	i.p.	Inhibition of CRC peritoneal metastasis and lysis of Peritoneal tumour cells	(15)
Reovirus	Pelareorep	i.t.	Lysis of KRAS-mutated CRC cells	(16)
	Pelareorep	Cell experiment	Promotes immune-mediated recognition and destruction of tumour cells	(17)
	RC402	p.o.	Significantly inhibits the growth of colorectal cancer tumours (combination with anti-PD-1 blockade)	(31)
HSV	Δ47/Δ34.5/IL12 HSV-1	Cell experiment	Effective in killing CRC cells	(18)
	oHSV-2	i.t.	Increased infiltration of immune cells	(19)
Adenovirus	SPDD-UG	i.t.	Inhibits CRC cell growth and induces apoptosis in CRC cells	(22)
	rAd.DCN.GM	i.t.	Significantly inhibits CRC tumour growth and distant metastasis	(23)
	CD55-TRAIL	Cell experiment/i.t.	Effective antitumour effects on CRC cells in vitro and in vivo (combination with luteolin)	(24)
Measles virus	MeVac FmIL-12	i.t.	Effective induction of apoptosis in CRC cancer cells	(26)
ORFV	NA1/11	i.t.	Inhibition of CRC growth and metastasis by induction of CRC apoptosis	(27)
VSV	M51R VSV	i.p.	Inhibited the growth of PSD in CRC	(28)
CVB3	PD	Cell experiment/i.t.	Efficient infection and lysis of CRC cells	(29)
	PD-H	i.t.	Effective tumourolytic activity against CRC	(30)

## Current status of immune checkpoint inhibitors for colorectal cancer

Immune checkpoints are cell-surface proteins whose function is to control the initiation, duration, and magnitude of the immune response. Disruption of immune checkpoints is one way to inhibit tumor immune evasion and stop cancer progression. The two most clinically relevant checkpoints, CTLA4 and PD-1, act as brakes on the anti-cancer immune response. Therefore, by inhibiting PD-1 or CTLA4 checkpoints or simultaneously inhibiting PD-1 or CTLA4 checkpoints, tumor-specific T cells can be expanded and stimulated to perform anti-tumor functions (32).

Immune checkpoint inhibitors (ICIs) increase T-cell activity and the ability to kill tumor cells by inhibiting these immune checkpoint activities. At present, ICIs have been shown to have a considerable improvement in the prognosis of patients with microsatellite instability-high (MSI-H)/DNA mismatch repair (dMMR) deficient (MSI-H/dMMR) tumors. In parallel, a series of clinical trials have demonstrated that patients with MSI-H/dMMR mCRC can be treated with Pembrolizumab and nivolumab monotherapy or nivolumab combined with Ipilimumab (33).

### PD-1/PD-L1 inhibitors

Programmed cell death-1 (PD-1) is a transmembrane glycoprotein expressed in activated T cells, and programmed cell death ligand 1 (PD-L1) is expressed on antigen-presenting cells and tumor cells. In the tumor microenvironment, the release of cytokines (IFN-γ, ILs, TNF-α) can induce high levels of PD-L1 expression. At the same time, the binding of PD-1 to PD-L1 can induce tumor cells to evade immune surveillance by inhibiting T-cell activation and proliferation (34). Therefore, PD-1/PD-L1 inhibitors can restore the activity of T cells and their ability to kill tumor cells by blocking PD-1/PD-L1 signaling for therapeutic purposes (35).

Currently, the main PD-1 inhibitors used in clinical studies for treating colorectal cancer are Pembrolizumab and Nivolumab. Pembrolizumab is currently approved by the US Food and Drug Administration (FDA) for the first-line treatment of unresectable or metastatic colorectal cancer with high microsatellite instability or mismatch repair defects (36).

A KEYNOTE-164 clinical phase II study enrolled 124 patients with MSI-H/dMMR CRC receiving≥2 standard treatments. The results showed that in cohort A (fluoropyrimidine, oxaliplatin, and irinotecan in combination or not with anti-vascular endothelial growth factor/epidermal growth factor receptor monoclonal antibody), the ORR was 33%, including 2 CRS; In cohort B (≥ 1 previous treatment), the ORR was 33%, including 5 CRS. This study confirms the anti-tumor effects and clinical efficacy of Pembrolizumab in previously treated MSI-H/dMMR colorectal cancer patients (37). In the phase 3 KEYNOTE-177 trial, 307 MSI-H/dMMR CRC patients were enrolled in the study and randomly assigned to receive either Pembrolizumab or chemotherapy and 294 MSI-H/dMMR CRC patients (152 treated with Pembrolizumab and 142 with chemotherapy) were analyzed by HRQOL. These data further confirm the benefit of Pembrolizumab as a first-line treatment for patients with MSI-H/dMMR CRC (38).

Growing tumors show one of three patterns: (1) little or no tumor-infiltrating immune cell infiltration (“immune ignorance “). (2) the presence of intra-tumoural immune infiltration with minimal or no PD-L1 expression (“non-functional immune response”). (3) Immune infiltration that resides only around the outer edge of the tumor cell mass (“excluded infiltrate “). However, most treatment patients showed a lack of PD-L1 upregulation in tumor cells or tumor-infiltrating immune cells (39). Therefore, most cancer patients are resistant to PD-1/PD-L1 blockade. The presence of an “ immune brake” on activated T cells ensures that they will only mount an effective cytotoxic response if the antigenic stimulus is of sufficient strength or affinity and is perceived in the context of an appropriate pro-inflammatory signal (40). Therefore, there is an urgent need to find alternative immunotherapeutic and anti-tumor approaches to use in combination with PD-1/PD-L1 inhibitors to overcome the limitations of ICIs in treating tumors.

### CTLA-4 inhibitors

Cytotoxic T-lymphocyte antigen 4 (CTLA-4) is a member of the immunoglobulin-associated receptor family, and the binding of CTLA-4 to its ligand B7-2 protein inhibits T-cell proliferation activation and cytokine production while decreasing the immune response (41). Therefore, blocking CTLA-4 reactivates T cells and restores their ability to attack cancer cells. However, CTLA-4 blockers alone are ineffective in treating colorectal cancer. A clinical study showed that 47 pretreated mCRC patients treated with a CTLA-4 blocker (tremelimumab) failed to show clinically meaningful results after monotherapy (42). Therefore, combining CTLA-4 blockers with other drugs for colorectal cancer is a primary research direction.

Ipilimumab, a fully human immunoglobulin G1 monoclonal antibody capable of blocking CTLA-4, was approved by the FDA in July 2018 for the immune combination treatment of MSI-H mCRC after progression on standard chemotherapy. In a large combination immunotherapy study, the CheckMate-142 trial, 119 MSI-H/dMMR CRC patients were treated with Ipilimumab combined with the PD-1 inhibitor Nivolumab and had an investigator-assessed ORR of 55% (95% CI, 45.2-63.8) and a 12-week disease control rate of 80%. This demonstrates that Nivolumab and Ipilimumab synergistically promote T-cell anti-tumor activity through complementary mechanisms of action, which provides a clinical experimental basis for CTLA-4 inhibitors combined with PD-1 inhibitors in the treatment of MSI-H/dMMR mCRC (43). Therefore, this suggests a new strategy for the future treatment of colorectal cancer: through the complementary mechanism of dual inhibitors, block the inhibition of CTLA-4 on T cell activation in the early stage and PD-1 on T cell anti-tumor response in the later stage, improve the immune response and promote the anti-tumor immune response (44).

### Other potential immune checkpoint targets in CRC

So far, only immunotherapies with two checkpoint targets, CTLA-4 and PD-L1/PD-1, have been approved for clinical use. However, most cancer patients do not respond to treatment with PD-1/PD-L1 inhibitors or CTLA-4 inhibitors. Therefore, as research progresses, several promising novel immune checkpoint targets are emerging as breakthrough points for cancer immunotherapy.

T-cell immunoglobulin and mucin-containing structural domain protein 3 (TIM-3) is a suppressive immune checkpoint molecule. TIM-3 has a suppressive effect on T-cell-induced immune responses. Blocking TIM-3 reverses T-cell dysfunction and restores anti-tumor immunity. Co-blockade of TIM-3 and PD-1 improves anti-cancer T cell responses in patients with advanced cancer (45). Increased TIM-3 expression in tumor tissue of CRC patients was positively correlated with poor prognosis and tumor progression. TIM-3 expression on CRC-infiltrating T cells in TME was significantly higher than TIM-3 expression on T cells in circulation. Furthermore, in CRC, the majority of TIM-3 expressing T cells in TME co-express PD-1 (46). A related study analyzed the expression of PD-1 and TIM-3 in patients with surgically treated stage I-III CRC. They found that CRC patients with high PD-1 and high Tim-3 expression had a worse prognosis than CRC patients with single high or double low expression. Thus, TIM-3 is a crucial mediator of CRC progression and may be a potential independent prognostic factor for CRC patients (47). Co-expression of TIM-3 and PD-1 on T cells may lead to resistance to ICIs in CRC patients. Therefore, treatment targeting Tim-3 with anti-PD-1 or other immunotherapies may provide clinical benefits for CRC patients (48).

TIGIT (T-cell immunoglobulin and immunoreceptor tyrosine motif (ITIM) structural domain) is a novel immune checkpoint. TIGIT is expressed on CD4 and CD8 T cells as well as innate lymphocytes, including NK cells and γδ T cells (49). PD-1 and CTLA-4 are predominantly expressed by tumor-infiltrating T cells and rarely by tumour-infiltrating NK cells. Therefore, using PD-L1/PD-1 and CTLA-4 inhibitors is not beneficial for NK cell failure. In contrast, blockade of TIGIT has been shown to prevent NK cell depletion and promote NK cell-dependent tumor immunity. At the same time, TIGIT, as a monotherapy, may be better able to reverse the failure of both T and NK cells (50). It has now been demonstrated that TIGIT is up-regulated in colorectal cancer with infiltrating lymphocytes, including CD3, CD4, CD8, and NK cells. By secreting TGF-β1, colorectal cancer cells can up-regulate TIGIT expression, promote CD8T cell depletion and facilitate tumor immune escape (51). Furthermore, it was found that elevated TIGIT on CD3 T cells led to functional defects and impaired glucose metabolism and that blocking TIGIT restored CD3 T cell activity and inhibited tumor growth. Thus, blocking TIGIT and restoring T-cell metabolic activity may represent immunotherapy for CRC (52).

LAG-3 is an Ig-like structural domain type I transmembrane protein with four structural domains called structural domain 1 (D1) to structural domain 4 (D4). Currently, LAG-3 is a promising therapeutic target for cancer immunotherapy. LAG-3 acts similarly to PD-1 and helps tumor cells to undergo immune escape (53). One study found that blocking both LAG-3 and PD-1 promoted T cell-mediated immune responses leading to a significant delay in tumor growth compared to anti-PD-1 antibody or anti-LAG-3 (LBL-007) treatment alone in CRC model mice. Thus, anti-LAG-3 and anti-PD-1 antibodies showed synergistic anti-tumor activity in CRC model mice. Anti-LAG-3 with anti-PD-1 could be a promising combination strategy for immunotherapy of CRC (54).

Currently, after the research results of PD-1 inhibitors and CTLA-4 inhibitors in the treatment of CRC, novel immune checkpoints such as TIM-3, TIGIT, and LAG-3 are not only potential biomarkers for diagnosis, prognosis, and survival prediction of CRC patients but also the next breakthrough point in immunotherapy for CRC. There are significant interactions between multiple immune checkpoints in the pathogenesis of CRC. Blocking one immune checkpoint alone may result in compensatory upregulation of other checkpoints. Therefore, combined blockade of multiple immune checkpoints may be a new immunotherapeutic strategy for CRC patients (55).

## Mechanism of action of oncolytic viruses combined with immune checkpoint inhibitors

### Immune checkpoint inhibitors are less effective in treating ‘‘cold’’ tumors

The tumor microenvironment (TME) refers to the complex multicellular environment in which tumors develop and typically includes T and B lymphocytes, tumor-associated macrophages (TAM), dendritic cells (DC), natural killer (NK) cells, neutrophils, and bone marrow-derived suppressor cells (MDSC) (56). The production of pro-inflammatory cytokines and the level of T-cell infiltration will somewhat alter the ‘‘cold’’ (non-T-cell inflammation) versus ‘‘hot’’ (T-cell inflammation) nature of TME, which closely correlates with the suppressive or supportive nature displayed during tumor development. ‘‘hot’’ tumors are characterized by molecular markers of T-cell infiltration and immune activation. ‘‘hot’’ tumors have a higher response rate to immunotherapy, whereas ‘‘cold’’ tumors show a marked T-cell deficiency or rejection (57). Many malignant tumor cells exhibit ‘‘cold’’ tumors. In addition to fewer infiltrating lymphocytes in TME, they exhibit low expression of PD -L1 and primary histocompatibility complex class I (MHC I) (58). Compared to “hot’’ tumors, immune checkpoint inhibitors have a minimal effect in treating ‘‘cold’’ tumors. Conversion of ‘‘cold’’ tumors to ‘‘hot’’ tumors will to some extent, increase the sensitivity of immunotherapy with immune checkpoint inhibitors. At the same time, combining relevant TME modulation therapies with checkpoint blockade is most likely to provide additional clinical benefits for patients with specific malignancies (59).

### Oncolytic viruses sensitize ‘‘cold’’ tumors to immune checkpoint inhibitors by modulating TME

Oncolytic viruses are able to induce lysis of tumor cells, which releases tumor-associated antigens and neoantigens (TAAs and TANs) that can be captured by tumor-infiltrating antigen-presenting cells (APCs), resulting in increased T cell infiltration (60). Oncolytic viruses promote immunogenic cell death (ICD), leading to the release of danger-associated molecular patterns (DAMPs) such as calreticulin (CRT), high mobility group protein 1 (HMGB1), and ATP. At the same time, oncolytic viruses promote the maturation of DC cells and recruit immune effector cells to the vicinity of dead tumor cells, enhancing phagocytosis of tumor cells by local DCs and macrophages (61).

In addition to the release of DAMPs, OV-mediated cancer cell lysis is often associated with the release of various pathogen-associated molecular patterns (PAMPs), including viral components such as nucleic acids (DNA, dsRNA, ssRNA, and 5′-triphosphate RNA), proteins and capsid components. DAMPs and PAMPs are recognized by pattern recognition receptors on antigen-presenting cells (APCs) (such as DC and NK cells) and serve as “danger” and “eat me” signals. This signaling attracts more DCs to the TME, leading to increased recruitment and maturation of tumor-specific T cells into the TME (62). Thus, oncolytic viruses can activate innate and acquired immunity, increase T-cell infiltration and reverse the tumor microenvironment so that ‘‘cold’’ tumors can be effectively immunologically activated and transformed into ‘‘hot’’ tumors, thus facilitating the action of immune checkpoint inhibitors (63).

**Figure 1:**
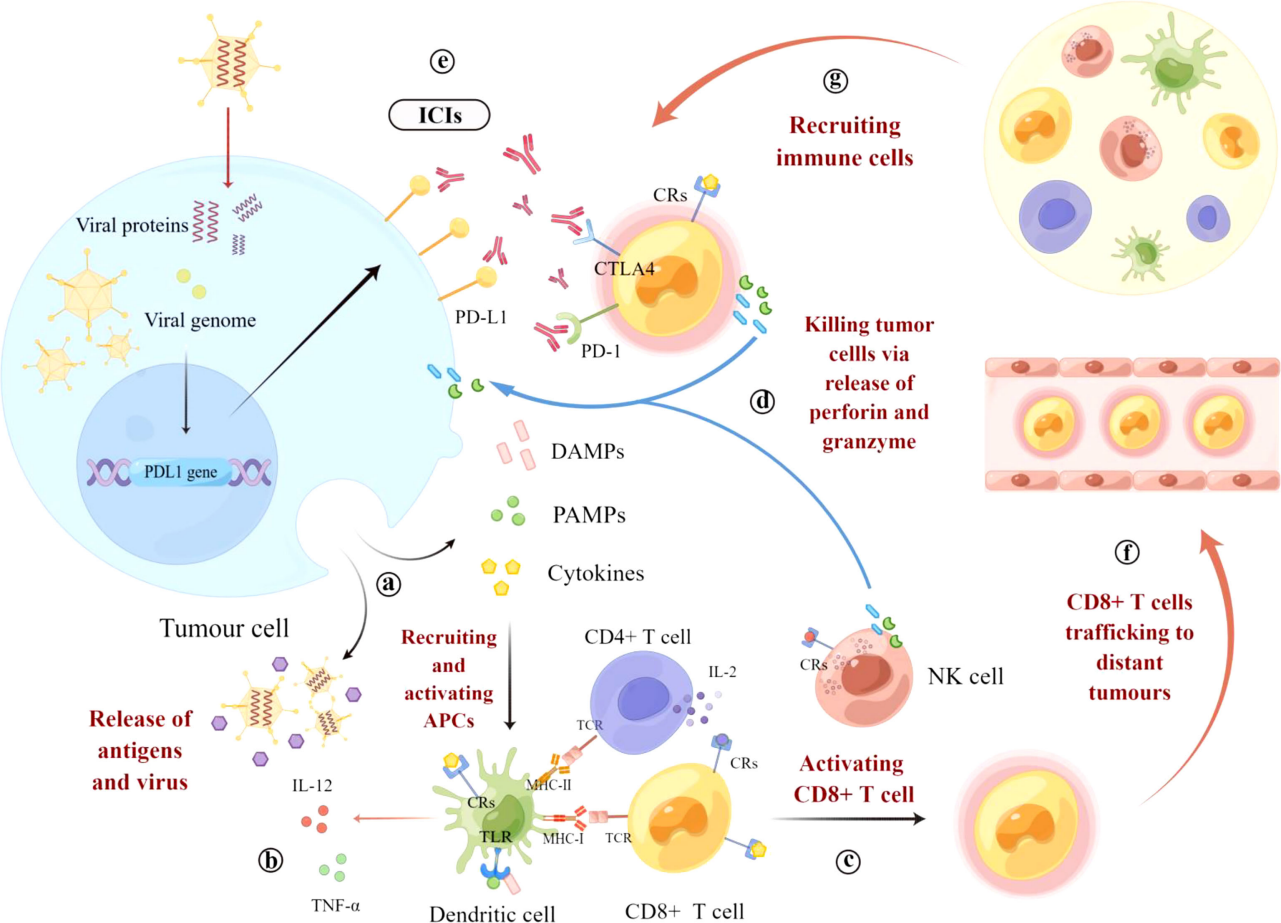
Mechanism of OVs combined with ICIs to stimulate anti-tumor immunity. **(A)** OVs enter tumor cells and undergo viral replication, leading to lysis and the release of danger-associated molecular pattern signals (DAMPs), pathogen-associated molecular patterns (PAMPs), tumor-associated antigens (TAAs), and pro-inflammatory cytokines. Viral progeny is also released, spreading to and infecting neighboring tumor cells. **(B)** These molecules recruit and activate antigen-presenting cells (APCs), such as dendritic cells (DCs), and promote the maturation of CDs through co-stimulatory markers while promoting the release of pro-inflammatory cytokines such as interleukin 12 (IL-12) and tumor necrosis factor (TNF-α) from CDs and recognition by cytokine receptors (CRs) on T cells and NK cells. **(C)** Mature dendritic cells cross-present antigens to CD4+ and CD8+ T cells *via* the major histocompatibility complex (MHC) and induce their expansion and activation. **(D)** T cells and NK cells eventually lyse tumor cells by releasing perforin, granzyme, and cytokines (IFN-γ, IL-2). **(E)** OVs infection leads to increased expression of immune checkpoint molecules such as PD-L1 and CTLA-4, thereby increasing the expression of the therapeutic targets of ICIs and sensitizing OV-infected tumor cells to ICIs. **(F)** In addition, local injection of OVs into individual tumor sites induces a distant effect, causing T cells to migrate to the site of metastatic disease, recognizing and killing distant tumor cells. **(G)** Cytokines and chemokines released in the tumor microenvironment can recruit immune cells for concerted anti-tumor activity.

A clinical trial using transgenic herpes simplex virus type 1 (Talimogene laherparepvec) in combination with pembrolizumab in patients with advanced melanoma showed that OVs might make tumor cells more sensitive to immune checkpoint inhibitors by up-regulating PD-L1 expression (64). In addition, local injection of OVs into individual tumor sites induces a distant effect known as the “peritoneal effect”. In this effect, distant uninfected tumors also experience immune-mediated rejection, inducing an inflammatory immune infiltrate (65). Thus, OVs and immune checkpoint inhibitors have complementary mechanisms of anti-tumor immunity, and the high selectivity of oncolytic viruses for tumor cells may lead to the local production of immune checkpoint inhibitors, thus providing a better safety profile for systemic administration (66) **(** **Figure 1** **).**

### “Armed” recombinant oncolytic viruses enhance the efficacy of immune checkpoint inhibitors

To date, oncolytic viruses as monotherapy have provided only modest anti-tumor effects in patients. Therefore, in order to increase immune stimulation, OVs have been designed to provide therapeutic transgenes to “armed” oncolytic viruses (67). OVs can be used as a therapeutic platform by inserting transgenes into the oncolytic virus genome through viral genome engineering techniques to manipulate its structure to create a characteristic “armed” OVs. These “armed” OVs with immunostimulatory or anti-cancer transgenes can be used as monotherapy or in combination with other therapeutic modalities to reduce side effects and improve anti-tumor efficacy. If an “armed” OV is designed for systemic delivery, it could be one of the options for treating patients with metastatic tumors (68). Currently, the primary use is to genetically engineer oncolytic viruses to carry PD-1/PD-L1 antibody genes or otherwise “armed” them to enhance the sensitivity of tumor cells to ICIs.

### “Armed” oncolytic viruses are carrying PD-1/PD-L1 antibody gene

Researchers built an “armed” oncolytic vaccinia virus (VV-iPDL1/GM) co-expressing PD-L1 inhibitor and GM-CSF. They found that intra-tumor injection with these engineered OVs promoted tumor infiltration of neoantigen-specific T cells while activating immune cells. VV-iPDL1/GM-secreted PD-L1 inhibitors inhibited neoantigen presentation of PD-L1 on tumor cells, leading to the systemic rejection of both the treated tumor and distant tumors. Therefore, using “armed” OVs co-expressing PD-L1 inhibitors and GM-CSF alone or combined with immune checkpoint inhibitors is a new therapeutic option for treating patients resistant to PD-1/PD-L1 blockade therapy (69). An M51R mutant vesicular stomatitis virus (VSV) has been engineered to express a single-chain antibody Fv fragment (scFv) encoded by the PD-L1 targeting antibody avelumab, a human IgG1 antibody. This novel OV effectively inhibited tumor growth in a mouse model. Thus, recombinant OVs expressing PD-1/PD-L1 antibodies are a promising agent for cancer therapy (70).

In a study, researchers encoded a humanized anti-PD-1 monoclonal antibody (anti-PD-1 monoclonal antibody) and designed a novel herpes simplex virus (HSV-aPD-1). Data from this experiment suggest that HSV-aPD-1 further improves the immune microenvironment and plays a crucial role in treating tumors that are not sensitive to ICIs or other immunotherapies (71). In addition, related studies have developed bioengineered cell membrane nanobubbles (PD1-BCMNs) with PD-1 to carry oncolytic adenovirus virus (OA). PD1-BCMNs-OA can effectively activate tumor-infiltrating T cells and trigger a robust anti-tumor immune response. Thus, PD1-BCMNs-OA provides a clinical rationale for combining oncolytic virus therapy with immune checkpoint inhibitors (72).

### “Armed” oncolytic virus enables enhanced anti-PD-1 therapy

The degree of inflammation in the tumor microenvironment affects the overall and progression-free survival rates. “Armed” OVs carrying the gene encoding inflammatory cytokines can further modify the tumor microenvironment. A study showed that a recombinant oncolytic poxvirus virus (hIL-7/mIL-12-VV) dually expressing IL-7 and IL-12 completely altered the tumor immune microenvironment by enhancing the inflammatory immune state, showing beneficial systemic anti-tumor efficacy, significantly increasing the sensitivity of solid tumors to systemic anti-PD-1 and anti-CTLA4. This combined IL-7 and IL-12 viral therapy could provide clinical benefit as a single therapy and has the potential to be effectively combined with immunotherapy for various types of solid tumors (73).

The anti-tumor efficacy of an engineered recombinant oncolytic herpes simplex virus (ONCR-177) with five transgenes was enhanced by systemic anti-PD-1 treatment. Experimental data showed that the RR of contralateral tumors treated with combination therapy was significantly enhanced by 40% RR compared with oncr-171 or anti-PD-1 monotherapy. Thus, ONCR-177 enhances tumor lysis tolerance and anti-tumor activity through activation of systemic immunity triggered by transgene expression and can be further enhanced by co-treatment with immune checkpoint inhibitors (74). It has been found that combined treatment with locally “armed” oncolytic adenovirus virus type 5 (ZD55-IL-24) and systemic PD-1 blockade resulted in synergistic suppression of locally and distantly established tumors. Local treatment with ZD55-IL-24 could help PD-1 blockade overcome the relatively low limitations of tumor immune infiltration and recognition. However, as ZD55-IL-24 must currently be administered intratumorally, it is only indicated for treating those few tumors with visible lesions such as melanoma and is challenging to use for the vast majority of tumors in clinical practice. Nonetheless, this study provides a viable experimental basis for the combination therapy of “armed” OVs (75).

In addition, a novel PD-L1 ICI with a cross-hybrid Fc region-mediated effector mechanism of IgG and IgA was designed. It is cloned as a conditionally replicating adenovirus (Ad-Cab) to limit virulence and release only into the tumor microenvironment. Ad-Cab secretes a cross-hybrid IgGA Fc fusion peptide that binds to PD-L1 binding and activates multiple immune pathways. Therefore, designing a novel ICI and expressing it with an oncolytic adenovirus could enhance tumor-killing efficacy while maintaining safety (76) **(** **Table 2** **)**.

**Table 2:** Modification and effects of “armed” oncolytic virus.

Modification type	“Armed”OVs	Modified features	Effect	References
Carries the PD-1/PD-L1 antibody gene	VV-iPDL1/GM	Co-expression of PD-L1 inhibitor and GM-CSF	Enhanced PD-1/PD-L1 inhibitor sensitivity	(69)
	VSV^M51R^-PD-L1	Expression of a single-chain antibody Fv fragment encoded by the PD-L1-targeting antibody avelumab	Effective inhibition of tumour growth	(70)
	HSV-aPD-1	Encoding humanized anti-PD-1 monoclonal antibody	Improving the immune microenvironment to increase susceptibility to ICIs	(71)
	PD1-BCMNs-OA	Bioengineered cell nanomembranes carrying PD-1	Effective activation of tumour-infiltrating T cells to increase anti-tumour immune response	(72)
Carriage of other genes enables enhanced anti-PD-1 treatment	hIL-7/mIL-12-VV	Dual expression of IL-7 and IL-12	Enhancing inflammatory response to alter TME to improve anti-PD-1 and anti-CTLA-4 sensitivity	(73)
	ONCR-177	Carries five transgenes: IL12, FLT3LG, CCL4, anti-PD-1 and anti-CTLA-4	Activating systemic immunity to enhance anti-PD-1 therapy	(74)
	ZD55-IL-24	Insertion of the anti-tumour gene mda-7 and IL-24 gene	Increasing tumour immune infiltration to enhance anti-PD-1 efficacy	(75)
	Ad-Cab	Cloning from a novel PD-L1 ICI with a cross-hybrid Fc region of IgG and IgA	Activates multiple immune pathways to kill tumour cells	(76)

## Application of oncolytic virus combined with immune checkpoint inhibitor in the treatment of colorectal cancer

Using ICIs for MSI/dMMR mCRC is a significant breakthrough in oncology. Unfortunately, the majority of mCRC patients are microsatellite stable (MSS)/DNA mismatch repair specialists (pMMR) (MSS/pMMR), and ICIs have not shown any clinical benefit in treating MSS/pMMR mCRC (77).

The oncolytic virus can enhance the response rate of tumors to immune checkpoint inhibitors. It is an ideal candidate for use in combination treatment strategies. Therefore, immune checkpoint inhibitors combined with the oncolytic virus may be a potential therapeutic option for treating MSS/pMMR mCRC.

Currently, experimental studies have shown that the use of oncolytic virus combined with immune checkpoint inhibitors has a better therapeutic effect on colorectal cancer. In an experimental study of triple immune therapy (combination of CSF-1R, OVs, and PD-1 antibodies) in a mouse model of CRC, 43% of the mice treated with the triple therapy achieved CR tumors and prolonged OS, and there was no tumor growth in 10 months from the implantation of CRC cancer cells to the end of the experiment. The researchers also observed a similar reduction in tumor load when T cells from surviving mice were treated with triple therapy and fed into the untreated CRC mouse model. Thus, this triple immune therapy therapeutic strategy could potentially overcome low T-cell infiltration and TAMs while overcoming the limitations of PD-1 immunosuppression. This could significantly improve the therapeutic efficacy of anti-PD-1 immunotherapy in colon cancer (7).

Researchers have established a telomerase-specific oncolytic adenovirus virus (OBP-301, telomerase) in which the human telomerase reverse transcriptase (hTERT) promoter element drives the expression of the viral E1A and E1B genes. This genetic modification allows OBP-301 to replicate in tumor cells and induce tumor cell-specific death selectively. In parallel, the researchers developed a CT26 *in situ* rectal tumor model with liver metastases, which was injected intra-tumorally with OBP-502 and treated in combination with PD-1 Ab. The results showed that this combination therapy inhibited liver metastases and rectal tumors. Thus, telomerase-specific oncolytic adenovirus virus led to a celiac effect by activating a systemic anti-tumor immune response, and the combination with PD-1 Ab produced a synergistic anti-tumor effect, even leading to tumor eradication (78).

In addition, another trial further enhanced the sensitivity of immune checkpoint inhibitors in a mouse model of dMMR CRC by using a combination of low-dose mitomycin C (mitomycin) and oHSV. It was also found that this treatment strategy promoted the infiltration of activated conventional dendritic cells type 1 (cDC1s) into the tumor (79).

The most common route of oncolytic virus therapy is intra-tumor or intravenous administration, but its use in clinical practice is limited. Researchers have developed an orally available oncolytic reovirus, RC402, which breaks through the limitations of the use of lytic viruses in clinical treatment. Oral RC402 monotherapy significantly increased CD8+ cytotoxic T cells and decreased CD4+CD25+Foxp3+ regulatory T cells in distant colon cancer. At the same time, the combination of RC402 and PD-1 inhibitor further inhibits colon cancer growth and enhances anti-tumor immunity within the tumor microenvironment, leading to complete tumor regression. Therefore, combining RC402 with PD-1 inhibitors could maximize the efficacy of PD-1 immune checkpoint blockade in colon cancer and trigger an effective anti-tumor immune response (31).

**Figure 2:**
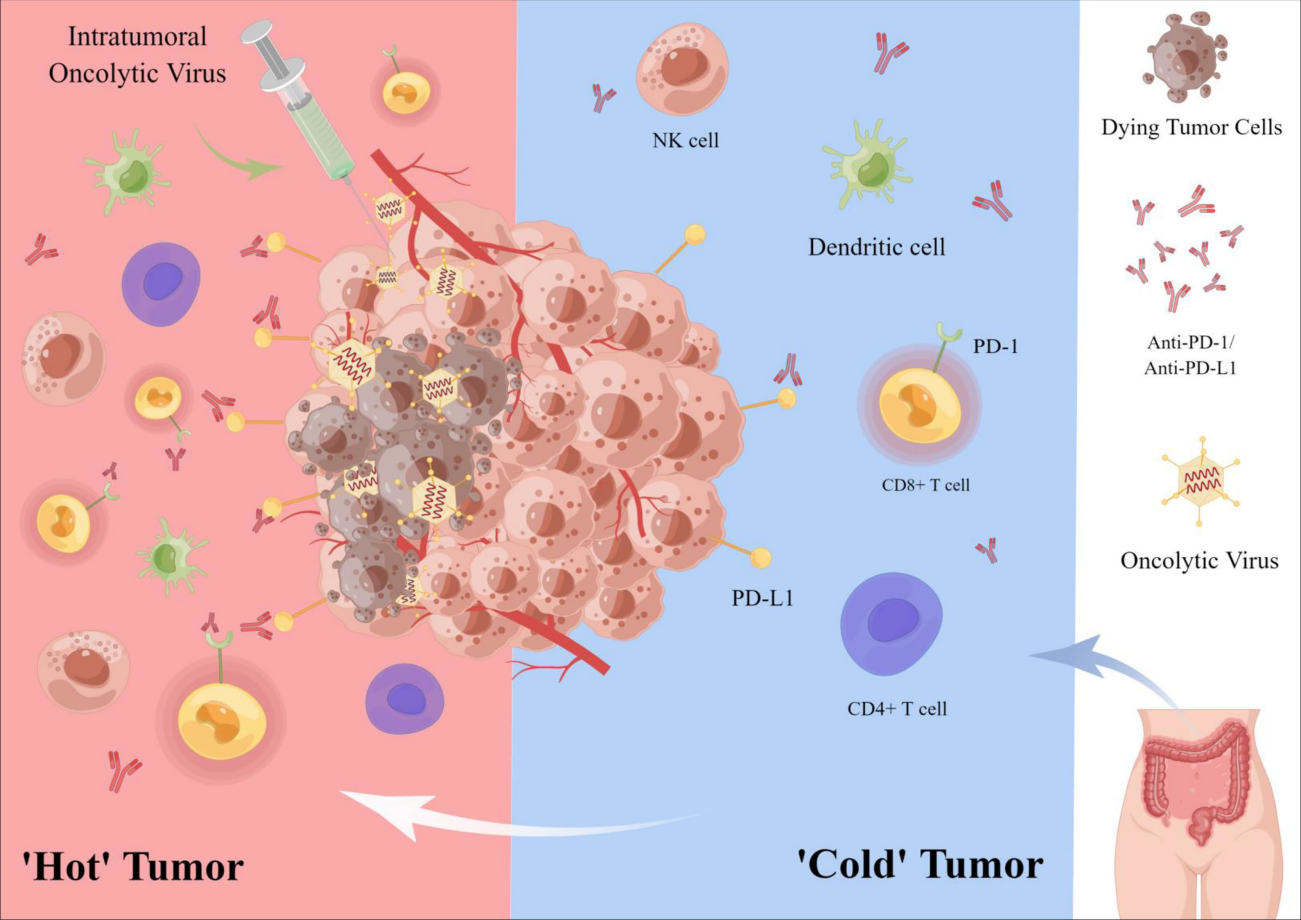
Oncolytic virus combined with immune checkpoint inhibitor in the treatment of colorectal cancer. OVs, lyse tumor cells while attracting immune cells (e.g., CD8+ T cells, NK cells) to the tumor microenvironment, thus transforming a ‘cold’’ tumor into a ‘hot’’ one to enhance the sensitivity of tumor cells to ICIs. In addition, OVs can enhance the targeted therapeutic effect of PD-1/PD-L1 inhibitors by up-regulating the expression of tumor cell immune checkpoint PD-L1.

In a clinical trial, 15 patients with refractory colorectal cancer received intravenous oncolytic vaccinia virus Pexa-Vec (pexastimogene devacirepvec; JX-594) every 14 days. This is the first treatment for patients with refractory, metastatic colorectal cancer using repeated doses of Pexa-Vec and administered by intravenous infusion. Ten patients (67%) had imaging stable disease. This clinical trial showed safety against the vaccinia virus in patients given repeated intra-tumor doses every two weeks (80). In CRC peritoneal metastases, the efficacy of ICI monotherapy in peritoneal tumors and malignant ascites is extremely low. In contrast, intraperitoneal oncolytic vaccinia virus (JX-594) induced an intense infiltration of CD8 T cells into peritoneal tumors, reversing intraperitoneal TME and reprogramming peritoneal tumors into T-cell inflamed tumors. This study showed that when JX-594 was combined with a PD-1 inhibitor, it was able to trigger effective anti-cancer immunity and eliminate peritoneal metastases in colon cancer, allowing better control of peritoneal metastases and malignant ascites in advanced colon cancer. Therefore, combining JX-594 with ICI (anti-PD-1, anti-PD-L1, or anti-LAG-3) is a promising strategy for treating peritoneal metastases from CRC (15) **(** **Figure 2** **)**.

## Conclusions and challenges

In recent years, immune checkpoint inhibitors (ICIs) have become a promising strategy for treating tumors. However, in treating colorectal cancer (CRC), ICIs have shown promising results only in MSI/dMMR CRC and are very limited for treating pMMR/MSS/MSI-L mCRC. Oncolytic viruses can directly lyse tumor cells and increase T-cell infiltration by activating immunogenic cell death (ICD) and releasing danger-associated molecular patterns (DAMPs) to recruit and promote DC maturation. At the same time, OVs promote the action of immune checkpoint inhibitors by modulating the tumor microenvironment (TME) and transforming ‘cold’’ tumors into ‘hot’’ ones. Thus, combining oncolytic virus therapy with immune checkpoint inhibitors may break through the limitations of ICIs treatment MSS/pMMR mCRC and enhance the sensitivity of colorectal tumor cells to ICIs.

Currently, various oncolytic viruses such as vaccinia virus (VV), reoviruses, herpes simplex virus (HSV), and adenovirus have been widely used in experimental studies of colorectal cancer. Studies have demonstrated that OVs can inhibit the growth and distant metastasis of colorectal cancer cells by lysing CRC cells, inducing apoptosis, and reversing TME, and have shown exciting results in treating CRC peritoneal metastases and KRAS-mutated colorectal cancer. The combination of OVs and ICIs has become the most promising strategy for treating solid tumors today. Many researchers have used OVs as a therapeutic platform to achieve better therapeutic results, using viral genome engineering techniques to make OVs carry PD-1/PD-L1 antibody genes or other therapeutic genes, creating “armed” OVs. These “armed” OVs have a complementary mechanism to ICIs in terms of anti-tumor immunity, enhancing the targeted delivery of OVs, further increasing T-cell infiltration, and providing comprehensive regulation of TME and the immune system resulting in a more substantial anti-tumor immune response rate. Therefore, combining “armed” OVs and ICIs may be one option for treating pMMR/MSS/MSI-L mCRC.

However, although the combination of OVs and ICIs has shown therapeutic efficacy in animal models of CRC, the specific treatment and prognosis of this combination therapy for CRC patients is unclear due to the lack of clinical trial data on OVs in combination with ICIs in CRC patients. As the immune response is a complex and highly regulated biological process, the efficacy of OVs in combination with ICIs in animal models of CRC cannot be fully replicated in patients with CRC. However, the results of several animal studies and clinical trials in other solid tumors suggest that OVs and ICIs combination therapy remains a very promising strategy in treating tumors. In addition, antagonism and the occurrence of immune-related adverse events (irAEs) need to be considered when combining OVs with ICIs.

At present, it remains challenging to get OVs to every primary and metastatic tumor site to achieve the desired effect. In distant metastatic lesions with low T-cell infiltration, the therapeutic effect of OVs combined with ICIs may be minimal. Therefore, there is a need to continue the search for a more accurate and effective immunomodulatory factor to control OV-mediated anti-tumor T-cell responses (81).

In conclusion, the treatment of colorectal cancer is limited by the limited treatment strategies available. Currently, the combination of oncolytic viruses and immune checkpoint inhibitors has shown promise in several clinical trials. This strategy is expected to be a promising treatment option for colorectal cancer. It is believed that in the near future, the combination of OVs and ICIs will bring hope to pMMR/MSS/MSI-L mCRC patients through clinical trial studies.

## Author contributions

Conceptualization: YR and X-BK; writing—review and editing: YR, J-MM, and Y-YW; visualization: ZF; supervision: X-BK and LY; project administration: GC. All authors have read and agreed to the published version of the manuscript.

## Funding

This research was funded by the Scientific research project of the Tianjin Education Commission, grant number 2021KJ134; Tianjin Municipal Education Commission Scientific Research Project, grant number 2019ZD11.

## Conflict of interest

The authors declare that the research was conducted in the absence of any commercial or financial relationships that could be construed as a potential conflict of interest.

## Publisher’s note

All claims expressed in this article are solely those of the authors and do not necessarily represent those of their affiliated organizations, or those of the publisher, the editors and the reviewers. Any product that may be evaluated in this article, or claim that may be made by its manufacturer, is not guaranteed or endorsed by the publisher.
